# Text4Support Mobile-Based Programming for Individuals Accessing Addictions and Mental Health Services—Retroactive Program Analysis at Baseline, 12 Weeks, and 6 Months

**DOI:** 10.3389/fpsyt.2021.640795

**Published:** 2021-05-28

**Authors:** Jasmine M. Noble, Wesley Vuong, Shireen Surood, Liana Urichuk, Andrew J. Greenshaw, Vincent I. O. Agyapong

**Affiliations:** ^1^Department of Psychiatry, Faculty of Medicine and Dentistry, University of Alberta, Edmonton, AB, Canada; ^2^Addiction and Mental Health, Alberta Health Services, Edmonton, AB, Canada

**Keywords:** mental health, intervention, technology, text-messaging, cognitive behavioral therapy, mobile, addiction

## Abstract

**Objective:** Where traditional approaches fall short, widely accessible and accepted, yet under leveraged, digital technologies such as text messaging present novel opportunities to solve a range of health care solutions. The following provides a preliminary analysis of the Text4Support program, a text-messaging intervention using the principles of cognitive behavioral therapy, which seeks to support the health and well-being of individuals seeking support for addiction or mental health concerns. The goal of this study was to assess whether the Text4Support program improved the perceived overall mental well-being of participants.

**Methods:** The evaluation analyzes survey responses of individuals who were enrolled in the Text4Support program beginning in July 2019, who had completed the 6-months program by May 2020. Participants were asked to provide responses to three surveys during their time in the program—at baseline, 12-weeks and 6-months, which included questions documenting demographic information, general satisfaction with the program, and a participants' level of “global distress” through use of the Clinical Outcomes Routine Evaluation System (CORE-10)—a validated brief 10-item assessment and outcome measurement tool used to assess conditions including anxiety, depression, physical problems, and risk to self.

**Results and Conclusions:** This data set did not include a large enough sample of participants to reach statistical significance. Nevertheless, the study provides some preliminary analysis, and identifies opportunities for the future analysis and research.

## Introduction

Global mobile phone ownership has proliferated and presents an unprecedented opportunity to bring substance use and mental health related support services to more individuals in new and innovative ways, regardless of where or when they need them. It is estimated that there are over 8 billion mobile phone subscriptions worldwide (2019) (1, 2). Nationally, 90% of Canadians are estimated to have a mobile phone (up 1.8% from 2016/17) (3). Additionally, mobile service coverage is expansive, with 99.4% of the Canadian population estimated to have mobile coverage, including 98.0% of rural communities and 88.2% of First Nations reserves (3).

Although substance use and mental illness are the leading causes of disability in Canada, a third of Canadians over the age of 15 years indicate that they do not feel their mental health needs are being adequately met (4), and only half of those experiencing a major depressive episode claim to have received adequate care (5). In Alberta, a similar observation is made, with 49% of Albertans indicating that they did not receive adequate services to address their mental health needs (6). The cost of the Canadian mental health burden is high, estimated at $51B CAD annually including lost productivity, health care costs, and decreases in quality of life (7).

In 2019, an outbreak of a disease called COVID-19 (virus name: SARS-CoV-2) resulted in a global pandemic. As of February 28, 2021, COVID-19 has spread globally with 114 million known cases, and 2.53 million deaths. In anticipation of a high volume of serious hospitalizations with technical respiratory needs, Canadians have been asked to self-quarantine and/or practice social distancing in an effort to reduce the burden to health systems (8). Although research remains ongoing to document the realized impacts of COVID-19, respondents from multiple public surveys deployed during the pandemic have noted experiencing multiple mental health stressors, such as economic instability, fear of getting sick, and life disruption as a cause of the COVID pandemic, resulting in stress, anxiety and depression (9). In reflection of this and negative mental health outcomes observed in previous epidemics and pandemics (10–13), it is widely agreed by the medical community that there will be a wave of widespread need for mental health related services resulting from the pandemic (9).

Text messaging interventions using mobile phones and/or smartphones may present a cost-effective avenue to expand the range of individuals who can be reached with mental health services. Whether they live in dense urban zones or remote rural regions, this form of intervention may allow for the bridging of systems of care, increasing access to services for those who need it. Text messaging capabilities are considered standard and already embedded on most mobile phones (14–16), allowing researchers and health care administrators the opportunity to tap into pre-existing infrastructure and networks. In consideration of the simplicity of the skill set required, individuals in possession of a mobile phone are generally literate in the sending and receiving of text-messages. Furthermore, text-messaging interventions have been subject to numerous evaluations and show considerable promise (17–28). Unfortunately, existing addiction and mental health treatment and programming in Alberta is not accessing this opportunity, with only 10–40% of programs utilizing phones, and 2–7% utilizing the internet (6). Additionally, communities in more remote areas with less access to resources (6) typically experience higher prevalence rates of substance use and mental illness. As a result, patients often need to travel extensive distances to access care, which in turn reduces the likelihood that they will seek care when needed (6).

Cognitive Behavioral Therapy (CBT) is a form of psychotherapy which seeks to employ its user to challenge negative thought patterns in order to reduce negative behavioral patterns and address or treat specific psychiatric symptoms or disorders such as depression and/or generalized anxiety disorder (29). In reflection of its use without necessity for an in-person professional counselor, the use of cognitive behavioral therapy (CBT) has been explored via text-messaging as an intervention tool, with interest in elevating an individual's mood, and were found to be effective (30–36).

This study evaluates a new text messaging program called Text4Support, launched in late 2018 by a Canadian provincially based public health delivery system called Alberta Health Services (AHS) at two service locations in Edmonton, Alberta. The Text4Support programme allows subscribers to receive daily supportive text messages, which were pre-written by a committee of cognitive behavioral therapists and mental health professionals in collaboration with mental health service users. More specifically, the program fundamentally seeks to influence the mood of participants with depression and/or other mental health problems through use of positive reinforcement (via text messages) in order to correct or alter negative thought patterns. The lack of timely services, long wait times, transportation, and geographic barriers are often experienced by clients trying to access AMH care. As such, the goal of Text4Support is also to provide supplementary support for individuals waiting to access or are receiving AHS AMH services. Text4Support is not meant to replace AHS AMH services, but instead to complement existing services. Once registered, participants continue to receive text messaging regardless of if or when they receive access to other public health services.

This study is a retrospective analysis of data collected by the Text4Support implementation team. Data was collected for internal monitoring purposes to evaluate the Text4Support program. The study hypothesized that the Text4Support program would improve the perceived overall mental well-being of participants. Well-being is defined in this study as, “the presence of positive emotions and moods (e.g., contentment, happiness), the absence of negative emotions (e.g., depression, anxiety), satisfaction with life, fulfillment and positive functioning (37–41).” This was to be assessed through the analysis of participant responses to survey questions, namely via Clinical Outcomes Routine Evaluation System (CORE-10) questions, a validated brief 10-item assessment and outcome measurement tool used to assess “global distress” including anxiety, depression, physical problems, and risk to self. Secondary measures included participant satisfaction, comfort level with technology, existing support, and perceived barriers to care. This study began in July 2019, prior to the COVID-19 pandemic, and ended during the pandemic, in May 2020.

## Materials and Methods

### Study Design and Participants

This was a retrospective analysis of Text4Support, a program that delivers daily supportive text messages to participants' mobile phones over a 6-months period, with the option to opt out of the program at any time (more detail on the development and composition of text messages below). Participants were recruited from ACCESS 24/7 and ACCESS Open Minds clinics starting in July 2019, and enrolment remains ongoing. For the purposes of this analysis, data were extracted on participants who enrolled after the program began in July 2019 and had completed the program prior to May 15th, 2020 (i.e., enrolled by November 15, 2019). Individuals seeking access to mental health and addiction services were asked if they would like to participate in the Text4Support program when meeting with a physician or mental health therapist at the clinics. These individuals may have been seeking or actively engaged in addiction and mental health services, or seeking one-off support.

Study participants met the following inclusion criteria:

1. age 18 years and above;
2. had a mobile phone with text-messaging capabilities;
3. were generally familiar with text messaging technology (could successfully retrieve text messages); and,
4. were able to read English.

Please note: this study used the same inclusion and exclusion criteria as was used for participation in the Text4Support program itself.

If interested in the study, phone numbers from participants were logged into the Text4Support program system, and they were assigned a “theme” or “texting group” for their daily text messages at the discretion of the physician or mental health therapist. Themes were developed following the analysis of historical AHS aggregate service data, which provided information on commonly presented addiction and mental health concerns. This information was organized into eight major categories using participant friendly language (clinical language in parenthesis): anxiety; problematic elevated or irritable mood (bipolar disorders); unusual experiences (psychosis); difficulties managing emotions and relationships (personality disorders); depression; substance use; coping with major life events (adjustment disorders); and general well-being.

Individual daily text messages were then pre-written for each texting theme by cognitive behavioral therapists and mental health professionals in collaboration with mental health service users. Text message content included two dimensions: (1) general supportive content including messages of self-care, social support, hope, affirmation, and recovery; (2) theme specific content focused on the management of symptoms related to the specific categories outlined above (e.g., activity scheduling in depression). In the period of 6 months, a participant would receive 183 text messages, with no repetition; approximately 80% of messages were supportive content and 20% were theme specific.

Examples of text messages included the following:

- A healthy body can set the stage for a healthy mind. Do your best to maintain a healthy diet and try to exercise (general).
- Identify people in your life who you enjoy being with and who do not use substances. Try to spend more time with them (substance use theme).
- When you are feeling anxious, connect with your breath. Take 2 min to notice it flowing into and out of your body (anxiety theme).

Within 24 h of joining the program, participants received a text message with an online link to a consent form, as well as a baseline survey. After completion of the consent form, the participant then began receiving text messages. Additionally, participants received a follow-up survey at 12-weeks and 6-months marks to assess participant satisfaction and impact, and to monitor program effectiveness. Participants of the Text4Support program could end their participation at any time by texting “STOP” to the number through which they were receiving text messages. With permission from AHS, our study team extracted anonymized data from these surveys for analysis.

This study hypothesized that the Text4Support program would improve the perceived overall mental well-being of participants, assessed through the analysis of follow-up surveys provided by participants at baseline, 12-weeks, and 6-months marks of the program. The primary outcome measure for this study was the mean difference in participant self-reported improvement in overall mental well-being over the course of the intervention via responses to Clinical Outcomes Routine Evaluation System (CORE-10) questions—a validated brief 10-item assessment and outcome measurement tool drawn from CORE-OM, or Clinical Outcomes Routine Evaluation System Outcome Measure (“parent measure”). The CORE-OM and shortened CORE-10, are validated tools used to assesses “global distress,” covering anxiety, depression, trauma, physical problems, functioning and risk to self (42). Barkham et al. state the internal reliability (alpha) of the CORE-10 to be 0.90 and the score for the CORE-10 correlated with the CORE-OM at 0.94 in a clinical sample and 0.92 in a non-clinical sample (43).

Secondary outcome measures included Likert scale questions, asking participants to rate their level of satisfaction with the program, as well as comfort level with technology, and perceived levels of support. Additionally, participants were provided with a list of potential barriers to care and asked to indicate which they have experienced, inclusive of an open text option to collect barriers not otherwise mentioned. Please note that Likert scale questions in this study were derived by the study team, and were not a standardized tool.

With a prediction that daily supportive text messages would result in a 25% reduction in mean CORE 10 scores at 12 weeks and 6 months from baseline, a population variance of 5.0 for the CORE-10 mean score, a 1-sided significance level α = 0.05, and an acceptable difference between sample mean and population mean CORE-10 score of zero (μ-μ0 = 0), the estimate was that a sample size of 686 would be needed to detect mean differences between the baseline and the 12 weeks and 6 months mean CORE-10 scores with a power of 80% (β = 0.2).

Data were analyzed using Excel and IBM Statistical Package for Social Sciences (SPSS) Statistics for Windows version 24. Paired *t*-tests were used to assess differences between the mean CORE-10 scale scores at baseline and 12 weeks, 12 weeks and 6 months, and baseline and 6 months for subscribers who completed the instrument at both time intervals of interest.

The study protocol was approved by the Health Research and Ethics Board of the University of Alberta (Pro00086163).

### Consort Flow Diagram

Between July 17 and November 15, 2019, 310 potential participants were eligible to enter the study. Of those eligible, 296 gave consent to enter the study, and 25 declined, allowing for an enrolment rate of 95.5%. Fifty-two subscribers asked to stop receiving text messages after enrolment (dropouts), and 244 remained in the study for the full 6-months period, at a retention rate of 82.4%. Participants who dropped out were in the program for an average of 45.4 days.

The study enrolled 296 participants. This study remains ongoing, and further analysis is anticipated on a larger data set.

## Results

### Baseline Demographic and Clinical Characteristics

In Table 1, Text4Support participants who completed the program by May 15, 2020 (*n* = 296) were mostly female and had an average age of 36. A total of 143 participants completed the baseline survey, providing a completion rate of 48.3%. Respondents were primarily European/Caucasian in ethnicity, attained a high school or post-secondary education, and rented or owned a home. As noted, phone numbers from participants were logged into the Text4Support program system, and they were assigned a “theme” or “texting group” for their daily text messages at the discretion of the physician or mental health therapist. Almost half of participants were assigned into the depression texting group, followed by anxiety, and substance use.

**Table 1:** Baseline demographic characteristics of participants.

**Characteristics of study participants (*n* = 296)**	
**Characteristic**	**Value** ***n*** **(%)**
Gender^*^ (*n* = 296)	*n* (%)
Female	188 (63.5)
Male	108 (36.5)
Age group (years) (*n* = 296)	*n* (%)
18–24	71 (24.0)
25–34	96 (32.4)
35–44	55 (18.6)
45–54	39 (13.2)
55–64	26 (8.8)
65+	9 (3.0)
Ethnicity (*n* = 143)	*n* (%)
African/Caribbean	4 (1.4)
Asian	6 (2.0)
European/Caucasian	98 (33.1)
I do not know	3 (1.0)
Indigenous (i.e., First Nations, Métis, and Inuit)	9 (3.0)
Latin American	1 (0.3)
Middle Eastern	1 (0.3)
Unknown^**^	172 (58.1)
Prefer not to disclose	2 (0.7)
Education (*n* = 143)	*n* (%)
8th grade or less	2 (0.7)
High school	40 (13.5)
Unknown^**^	159 (53.7)
Post-secondary	53 (17.9)
Other	1 (0.3)
Some high school	15 (5.1)
Some post-secondary	22 (7.4)
Technical/trade school	4 (1.4)
Housing (*n* = 143)	n (%)
Couch surfing	1 (0.3)
Live with family or friends	35 (11.8)
Living rent free with partner	1 (0.3)
Low income housing	1 (0.3)
Unknown^**^	159 (53.7)
Own home	32 (10.8)
Rented accommodation	66 (22.3)
Shelter/street	1 (0.3)
Identified texting group (theme) (*n* = 296)	*n* (%)
Anxiety	82 (27.7)
Coping with major life events	9 (3.0)
Depression	131 (44.3)
Difficulties managing emotions and relationships	11 (3.7)
General well-being	5 (1.7)
Problematic elevated or irritable mood	14 (4.7)
Substance use	36 (12.2)
Unusual experiences	8 (2.7)

* *Please note that “gender diverse” and “if gender not listed, please specify:” were additional options provided to participants, however for this data set no one chose those options*.

** *Unknown is defined as participants who did not complete the baseline survey and/or who chose not to provide any answer to this specific question*.

To gauge level of support, participants were given 3 Likert scale questions inquiring into their levels of personal support (i.e., do you feel you have someone … to turn to for support when needed? You can trust and confide in? To go to for help in case of illness or disability?) and asked to rate their level of agreement with said statement, from “not at all” to “most of the time.” Of those who completed the baseline survey, 43.9% of respondents scored high (score of 11+), 38.8% moderate (score of 6–10), and 15.8% low (score of 1–5) support.

To measure level of comfort with technology, participants were also given 3 Likert scale questions asking their comfort level with mobile technology (i.e., I use mobile technology often; I am comfortable using mobile technology; I enjoy using mobile technology) and asked to rate their level of agreement with the statement, from strongly agree to strongly disagree. Of those who completed the baseline survey, 86% of respondents scored high (score of 11+), 9% moderate (score of 6–10), and 5% low (score of 1–5) in comfort level with mobile technology.

Individuals who dropped out of the study (i.e., texted STOP” to the Text4Support number) averaged 35 years old, and were also predominantly female (60%). For these participants, length of time in the program averaged 45.40 days with considerable variability (SD 51.61). The most frequent texting theme assigned to participants of the drop-out group was depression at 48%, followed by anxiety at 17%.

### Self-Reported Improvement

#### Clinical Outcomes Routine Evaluation Score

The primary outcome measure for this study is an analysis of the self-reported improvement in perceived overall mental well-being over the course of the intervention. This measure included the use of CORE-10. Table 2 summarizes participant CORE-10 scores at baseline to 12 weeks, 12 weeks to 6 months, and baseline to 6 months. CORE-10 scores are ranked as follows: 0–5 = healthy, 6–10 = low level, 11–15 = mild, 16–20 = moderate-to-severe, and 26+ = severe. Of those who completed the survey, participant clinical numeric scores generally saw an improvement of 22.79% from baseline to 12-weeks with a mean difference of −5.71 (95% CI = 2.03–2.74), and overall an improvement (baseline to 6-months) of 18.97% with a mean difference of −4.58 (95% CI = 2.85–3.57). There was no statistically significant change in the scores from 12-weeks to 6-months.

**Table 2A:** Self-reported well-being reported at baseline and 12 weeks.

**Measure**	***N***	**Baseline Scores | Mean (SD)**	**12-weeks scores | Mean (SD)**	**Mean difference (SD)**	**95% Confidence Interval**	**Df**	***t*-value**	***P*-value**	**Effect Size (Cohen's d)**
CORE-10	35^a^	23.74 (6.14)	18.34 (8.26)	−5.41 (6.84) −22.79%	2.03–2.74	34	4.67	<0.001	0.89

**Table 2B:** Self-reported well-being reported at 12 weeks and 6 months.

**Measure**	***N***	**12-weeks scores | Mean (SD)**	**6-months scores | Mean (SD)**	**Mean difference (SD)**	**95% Confidence Interval**	**Df**	***t*-value**	***P*-value**	**Effect Size (Cohen's d)**
CORE-10	13^b^	17.77 (6.17)	20.38 (8.16)	2.62 (7.15)	3.35–4.44	12	−1.32	0.212	−0.36

**Table 2C:** Self-reported well-being reported at baseline and 6 months.

**Measure**	***N***	**Baseline scores | Mean (SD)**	**6-months scores | Mean (SD)**	**Mean difference (SD)**	**95% confidence interval**	**Df**	***t*-value**	***P*-value**	**Effect size (Cohen's d)**
CORE-10	19^c^	24.47 (6.33)	19.89 (7.93)	−4.58 (7.85) −18.97%	2.85–3.57	18	2.54	0.02	0.64

*Higher CORE-10 Scores indicate greater level of distress*.

a *Includes individuals who participated in both baseline AND 12-week surveys*.

b *Includes individuals who participated in both 12-week AND 6-month surveys*.

c *Includes individuals who participated in Baseline AND 6 month surveys (may have missed 12 week survey) and those who participated in all 3 surveys*.

**Figure 1:**
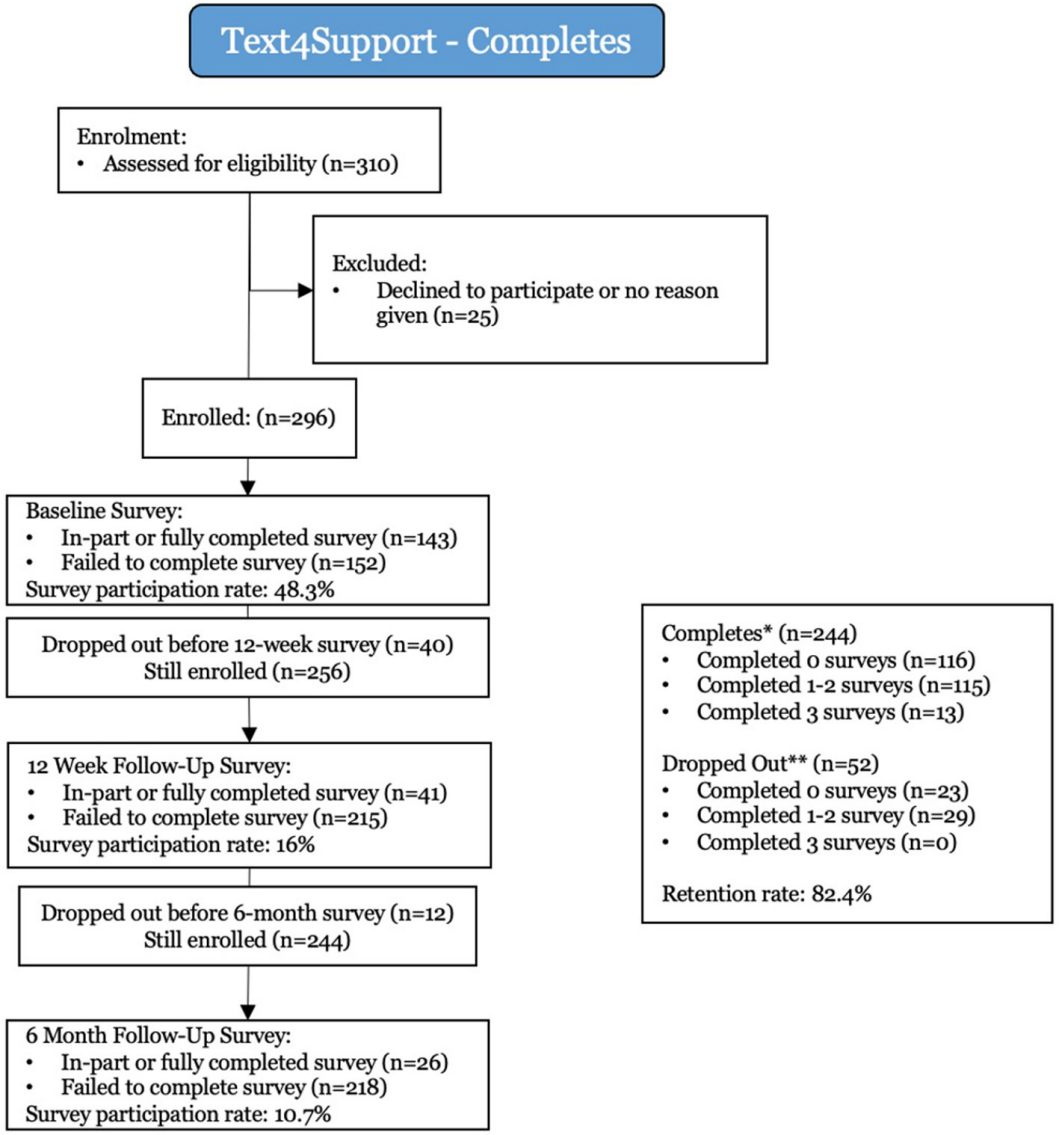
Consort Flow Diagram.

As Figure 1 depicts, participation rates for the baseline, 12-weeks, and 6-months surveys saw a reduction over time, from 48.3% completion rate for the baseline, to 16% survey completion at 12 weeks, and 10.7% completion at 6 months. The sample sizes for 12-weeks and 6-months surveys are thus low and lack statistical significance. Therefore, the results presented below are anecdotal observations, and more research is needed in order to draw any more formal conclusions. Additionally, at the time of survey completion, participants may have been admitted into a mental health program and/or provided with some form of mental health support, which may pose as a confounding variable and complicate analysis. Please see the discussion section below for further analysis on potential confounding variables.

#### Participant Satisfaction

Tables 3A,B provide participant satisfaction data for Text4Support participants from surveys administered at 12-weeks and 6-months. Participants in the Text4Support program mostly agreed that the Text4Support text messages were on topic, to the point, supportive, and positive for both the 12-weeks and 6-months surveys, respectively. A high percentage of participants indicated that they “always” or “mostly” read the text messages (98 and 96% for the 12-weeks and 6-months surveys, respectively).

**Table 3A:** Participant satisfaction with Text4Support - 12-weeks survey responses.

**Participant satisfaction with Text4Support−12-weeks (*n* = 42)**						
		**Strongly agree**	**Agree**	**Neutral**	**Disagree**	**Strongly disagree**
Text4Support text messages were:	On topic.	14 (35%)	22 (55%)	3 (8%)	1 (3%)	0 (0%)
	Not relevant to my concern.	1 (3%)	5 (13%)	8 (20%)	18 (45%)	8 (20%)
	To the point.	12 (30%)	22 (55%)	4 (10%)	1 (3%)	1 (3%)
	Not helpful.	0 (0%)	1 (3%)	5 (13%)	24 (62%)	9 (23%)
	Supportive.	18 (45%)	19 (48%)	3 (8%)	0 (0%)	0 (0%)
	Not encouraging.	0 (0%)	2 (5%)	4 (10%)	21 (53%)	13 (33%)
	Positive.	17 (43%)	22 (55%)	1 (3%)	0 (0%)	0 (0%)
	Negative.	0 (0%)	2 (5%)	3 (8%)	18 (45%)	17 (43%)
		**Always**	**Mostly**	**Sometimes**	**Rarely**	**Never**
How often did you …	Read the text messages?	32 (80%)	7 (18%)	0 (0%)	1 (3%)	0 (0%)
	Read the text messages and take action?	3 (8%)	11 (28%)	19 (48%)	6 (15%)	1 (3%)
		**Strongly agree**	**Agree**	**Neutral**	**Disagree**	**Strongly disagree**
Participating in Text4Support has helped me to …	Improve my quality of life?	4 (10%)	14 (35%)	18 (45%)	3 (8%)	1 (3%)
	Improve my overall mental well-being?	6 (15%)	20 (50%)	11 (28%)	2 (5%)	1 (3%)
	Improve my overall physical well-being?	3 (8%)	11 (28%)	16 (41%)	7 (18%)	2 (5%)
	Cope with loneliness?	5 (13%)	17 (43%)	14 (35%)	4 (10%)	0 (0%)
	Cope with stress?	4 (10%)	20 (51%)	11 (28%)	4 (10%)	0 (0%)
		**Yes**	**No**			
Are you currently accessing services for a.	Mental health concern(s)?	31 (79%)	8 (21%)			
	Substance use concern(s)?	3 (9%)	30 (91%)			–

**Table 3B:** Participant satisfaction with Text4Support−6-months survey responses.

**Participant satisfaction with Text4Support−6-months (*n* = 26)**						
		**Strongly agree**	**Agree**	**Neutral**	**Disagree**	**Strongly disagree**
Text4Support text messages were:	On topic.	10 (42%)	10 (42%)	3 (13%)	0 (0%)	1 (4%)
	Not relevant to my concern.	1 (4%)	2 (8%)	8 (33%)	10 (42%)	3 (13%)
	To the point.	11 (46%)	7 (29%)	5 (21%)	0 (0%)	1 (4%)
	Not helpful.	1 (4%)	0 (0%)	3 (13%)	14 (58%)	6 (25%)
	Supportive.	10 (42%)	12 (50%)	2 (8%)	0 (0%)	0 (0%)
	Not encouraging.	1 (4%)	0 (0%)	4 (17%)	11 (46%)	8 (33%)
	Positive.	13 (54%)	9 (38%)	2 (8%)	0 (0%)	0 (0%)
	Negative.	1 (4%)	0 (0%)	5 (21%)	7 (29%)	11 (46%)
		**Always**	**Mostly**	**Sometimes**	**Rarely**	**Never**
How often did you …	Read the text messages?	18 (78%)	7 (18%)	4 (17%)	0 (0%)	0 (0%)
	Read the text messages and take action?	3 (14%)	11 (28%)	9 (41%)	1 (5%)	2 (9%)
		**Strongly agree**	**Agree**	**Neutral**	**Disagree**	**Strongly disagree**
Participating in Text4Support has helped me to …	Improve my quality of life?	4 (17%)	10 (42%)	10 (42%)	0 (0%)	0 (0%)
	Improve my overall mental well-being?	5 (21%)	11 (46%)	8 (33%)	0 (0%)	0 (0%)
	Improve my overall physical well-being?	4 (17%)	5 (21%)	13 (54%)	1 (4%)	1 (4%)
	Cope with loneliness?	6 (25%)	7 (29%)	9 (38%)	2 (8%)	0 (0%)
	Cope with stress?	7 (29%)	11 (46%)	5 (21%)	1 (4%)	0 (0%)
		**Yes**	**No**			
Are you currently accessing addiction and mental health services for a …	Mental health concern(s)?	18 (75%)	6 (25%)			
	Substance use concern(s)?	3 (14%)	19 (86%)			
Overall participant satisfaction		12 weeks (*n* = 42)	6 months (*n* = 26)			
Using any number from 0 (not at all satisfied) to 10 (very satisfied), how would you rate your overall satisfaction with Text4Support?	Satisfied (8–10)	24 (60%)	15 (60%)			
	Neutral (5–7)	13 (33%)	8 (32%)			
	Dissatisfied (0–4)	3 (8%)	2 (8%)			

Similarly, a large majority of respondents said they read the messages and took action “always,” “mostly,” or “sometimes,” (84% at 12 weeks and 83% at 6 months), suggesting a high level of engagement with the texts which resulted in a behavioral response. For psychological support, most participants reported the service helped improve overall mental well-being, cope with loneliness, and cope with stress at the 12-weeks survey and 6-months surveys. Participants' responses were mixed for whether the messages improved overall physical well-being. Interestingly, at 6-months, participants mostly agreed that Text4Support improved their overall quality of life. Overall satisfaction for the program was high, with 60% ranking the program at between 8 and 10 out of 10 in both 12-weeks and 6-months surveys. As noted above, sample size for both surveys is insufficient to establish statistical significance, and further analysis on a larger data set is required.

#### Perceived Barriers to Care

Tables 4A,B present Text4Support participant perceived barriers of care, categorized by gender, ethnicity, housing, highest education completed, level of support, clinical scores, and texting group. These data are presented as percentages of participants who indicate that a particular item presents a barrier to them. Overall, “costs of services” is the barrier identified most frequently, with 44% of respondents identifying this as an issue. This is followed by “not knowing how to access services,” a barrier for 41% of respondents, and “not knowing what services are available” and “stigma with accessing services,” both identified as barriers by 37% of respondents.

**Table 4A:** Perceived barriers of care categorized by demographic characteristics of participants (in column %).

**Barrier**	**Total (%)**	***Gender***		**Age**			**Education**			**Ethnicity**				**Housing**		
		**F (*n* = 98)**	**M (*n* = 45)**	**19–34 (*n* = 92)**	**35–44 (*n* = 25)**	**45+ (*n* = 26)**	**High school or less (*n* = 57)**	**Completed or partial post-secondary (*n* = 75)**	Other^*^ (*n* = 5)	**European/ Caucasian (*n* = 98)**	Visible minority^**^ (*n = 12)*	**Indigenous (*n* = 9)**	**Unknown/ prefer not to say (*n* = 5)**	Stable^***^ (*n* = 133)	Unstable^****^ (*n* = 2)	**Other (*n* = 2)**
Access to childcare	7	10	0	9	8	0	11	5	0	6	0	33	0	8	0	0
Cost of services	44	44	44	50	36	31	42	51	20	49	42	22	20	46	50	50
Hour the services are available	18	22	9	20	12	19	23	16	0	22	8	11	0	18	50	50
I did not experience barriers	10	11	7	9	12	12	5	15	0	9	8	0	20	11	0	0
I do not know	9	9	9	9	4	15	12	8	0	4	42	11	20	8	50	50
Location of services	24	27	18	24	24	23	25	25	20	26	17	22	20	26	0	0
Not knowing how to access services	41	39	47	45	40	31	39	45	40	44	50	56	20	42	50	50
Not knowing what services are available	38	40	36	41	40	27	46	35	60	38	42	67	20	38	100	50
Stigma with accessing services	38	38	38	41	44	19	35	43	40	42	33	44	20	38	100	50
Transportation to services	19	19	18	22	12	15	30	11	20	19	17	22	20	20	50	0
Type of services available	17	19	13	17	24	12	25	13	0	22	8	0	20	17	100	0
Wait times to access services	31	33	27	33	36	19	37	29	20	34	33	22	20	31	100	50

* *Defined as trades training; realtor certification*.

** *Defined as African/Caribbean; Asian; Latin American; Middle Eastern*.

*** *Defined as rented accommodation; own home; live with family or friends*.

**** *Defined as couch surfing; shelter/street*.

**Table 4B:** Perceived barriers of care categorized by level of support, clinical support, and texting group identified (in column %).

**Barrier**	**Level of support**			**Clinical score**					**Texting group**							
	**High (*n* = 61)**	**Moderate (*n* = 54)**	**Low (*n* = 22)**	**Severe (*n* = 60)**	**Moderate-to-severe (*n* = 45)**	**Moderate (*n* = 22)**	**Mild (*n* = 12)**	**Low (*n* = 4)**	**Anxiety (*n* = 41)**	**Coping with major life events (*n* = 5)**	**Depression (*n* = 66)**	**Difficulties managing emotions and relation-ships (*n* = 6)**	**General well-being (*n* = 3)**	**Problematic elevated or irritable mood (*n* = 10)**	**Sub-stance use (*n* = 9)**	**Unusual experience (*n* = 3)**
Access to childcare	7	9	5	7	4	14	8	0	12	0	8	0	0	0	0	0
Cost of services	43	52	41	58	31	45	25	25	41	40	52	33	0	40	33	33
Hour the services are available	13	19	36	25	9	23	17	0	17	0	20	33	0	20	22	0
I did not experience barriers	20	4	0	0	13	18	17	50	17	0	3	0	33	30	11	0
I do not know	5	9	18	12	11	5	0	0	7	0	14	17	0	0	0	0
Location of services	16	35	23	30	22	14	17	25	17	40	21	33	0	50	33	33
Not knowing how to access services	38	48	45	50	44	23	17	50	44	80	45	33	0	30	11	33
Not knowing what services are available	34	48	36	45	42	23	17	50	32	60	44	33	33	40	33	0
Stigma with accessing services	39	44	27	50	33	32	8	25	37	80	36	50	33	10	44	67
Transportation to services	18	19	27	30	9	9	17	25	24	0	12	17	0	30	44	33
Type of services available	18	15	27	22	11	23	17	0	20	20	15	50	33	0	22	0
Wait times to access services	26	41	27	40	31	23	8	0	29	40	30	67	0	30	33	0

Between females and males, women identify cost of services most frequently as a barrier to care, whereas men identify not knowing how to access services. Younger participants report cost more frequently as a barrier, whereas for older participants cost and not knowing how to access services are equally barriers. Amongst participants with high school education or less, not knowing how to access services is the primary barrier to care. Whereas, amongst individuals with higher education, cost of services is most frequently identified. When reviewing ethnicity, cost is most frequently identified by the ethnic majority (European/Caucasian), while not knowing how to access services was the most frequently cited barrier amongst non-Indigenous visible minority groups, and Indigenous participants report not knowing what services are available as the largest barrier. Individuals who report higher levels of social supports most frequently report cost as a barrier to care, while those with less support report not knowing how to access services more frequently. The most frequently noted barriers based on texting group was mixed amongst individuals in different categories.

## Discussion

The goal of this study was to assess whether the Text4Support program improved the perceived overall mental well-being of participants. This study is novel as it provides an evaluation of a new text-based intervention that has been recently implemented by a health system, through use of real-world data. There are several interesting observations that can be made in the analysis of this data, although due to low sample size, further research is needed for observations to be substantiated.

### Clinical Improvement

Data from this study suggests that participants within this intervention may have experienced an overall improvement in clinical wellness from baseline to 6 months. However, this program was offered to complement existing services, for which, based on responses on the 12-weeks and 6-months surveys, 79% (12-weeks) and 75% (6-months) of respondents indicated that they were currently accessing services for a mental health concern, and 9% (12-weeks) and 14% (6-months) for a substance use concern. This evaluation did not isolate for types of services and treatments that individuals may have been exposed to at the time of this intervention, for which may have contributed to clinical scores observed. This limitation equally applies to the deterioration observed in clinical scores between 12-weeks and 6-months surveys.

Of note is the outbreak of the COVID-19 pandemic, and its potential confounding influence on the clinical scores of individuals during their time in the Text4Support program. Reflecting on previous historical analysis on similar health crises, pandemics may contribute to negative emotions such as stress, sadness, or loneliness (12), as well as problematic drug and alcohol use (44). Additionally, this particular pandemic has already led to the proliferation of significant stressors amongst the general public including pessimistic national and global financial forecasts (45), as well as job loss (46, 47). Please see further discussion on COVID-19 below.

### Enrolment, Retention, and Satisfaction

The enrolment and retention rates for the Text4Support program were high at 95.5 and 82.4%, respectively. This may have been due to high user satisfaction as suggested from 12-weeks and 6-months surveys, where overall satisfaction for the program was ranked as high, with 60% rating the program between 8 and 10 out of 10 for both 12-weeks and 6-months surveys.

This high degree of self-reported user acceptability and retention has been reflected in other text messaging studies. For example, a systematic review examining text messaging to support the health of individuals with problematic drug and alcohol use found high rates of intervention acceptability (18). In a text messaging intervention study offered to two Northern Albertan communities (Grand Prairie and Fort McMurray) in 2016 (*n* = 4,111), the majority of participants who responded to follow-up surveys indicated that the messages “made them hopeful about managing issues in their lives, feel in charge of managing depression and anxiety, and feel connected to a support system” and generally improved their mental health well-being (27). This is consistent with other text messaging interventional studies to support mental well-being, including a text-messaging intervention which sought to support women through pregnancy, and a text intervention directed to impoverished women in Bangalore, where the majority of individuals in both studies indicated they felt support and reassurance from the messages received by the interventions (33–36).

### Engagement

Of interest is whether the intervention led to behavioral change. As noted, 84% (12-weeks survey) and 83% (6-months survey) of respondents indicated that they “always,” “mostly,” and “sometimes” read the messages and took action, which suggests that participants experienced a high level of engagement with the texts which resulted in a behavioral response. This is reflected in other studies, where text messaging has been found to increase appointment, pharmaceutical, and treatment adherence, reduce substance use, and promote abstinence and relapse prevention (17–26). Additionally, this finding has been observed in the management and treatment of other illnesses. A review analyzing text-message interventions as tools for behavioral change, found evidence in eight out of nine sufficiently powered studies that text messaging was found to be an effective tool for behavioral change in disease prevention (48–50) and management (23, 51–55), specifically in the management of weight loss, smoking cessation, and diabetes management.

Of the participants in this study, 75% of participants were 44 years and younger. Of those participants who answered the survey questions around comfort level with technology, 85% indicated a high level of comfort. When grouped by age, 88% of respondents aged 44 and younger indicated a high level of comfort and 4% a low level of comfort with tech, vs. 71 and 8% of participants 45 years and older, respectively.

Prenksy's concept of “digital natives,” (i.e., individuals with high exposure at an early age to technologies such as the internet, video games, mobile phones, and/or social media), vs. “digital immigrant,” (i.e., individuals without exposure in their childhood or youth to digital technology (e.g., pre-internet), and the gap between these groups has been analyzed extensively for its impact on technology uptake and engagement (56, 57, 57–59). Digital natives can be defined as individuals born after 1980 and, “surrounded by digital media and other digital technologies.” The argument then is that because older generations have less exposure to and/or need to interact with digital technologies than younger people, they face unique barriers in their ability to use and navigate them with ease, and thus resulting in the reduction of engagement or uptake, or excluding them all together (60–63).

Results from this study might suggest a technological divide between generations. If Prensky's hypothesis is true, in reflection that 70% of health and mental disorders typically begin in childhood and adolescence (64), and peak alcohol consumption rates are observed between the ages of 18 and 35 years (65), the potential openness of this cohort to engage with and provide health information, in addition to their familiarity with new technology as “digital natives” (66), presents a window of opportunity for prevention, early screening, and intervention.

It is important to note however, that critics to Prenksy's hypothesis argue that this divide in technological uptake in actuality cannot be generalized to simply a generational gap in exposure, but instead is complex and inclusive of many other social and environmental factors such as an individual's socioeconomic predicament, gender, rurality, cultural heritage, and patterns and/or obstacles of use (67, 68). Additionally, the technology or platform under analysis may also change the rates of technological uptake. This is evident when exploring the uptake of social media platforms (e.g., WeChat, Facebook, Twitter), which have been widely adopted in Canada [25.3 million estimated social network users in 2018 (69)], where membership is not restricted to younger demographics, but instead utilized generally by all age groups (70–72). The difference cross-generationally being instead in *how* individuals interact with the technology, rather than *whether* individuals used it at all (70, 73). In reflection that the penetrance rate for mobile phones in Canada is 90%, interventions reliant on the relatively rudimentary technology of text messaging may actually mitigate rather than magnify generational disparities in technology use. As noted, 85% of all respondents of the baseline survey indicated a high level of comfort with technology, including 71% of participants over the age of 45. This could suggest that older generations are generally comfortable with technology, and/or may present evidence of selection bias, where individuals who are comfortable with technology are generally more likely to participate in a study or program using technology as a medium for intervention/support.

The majority of participants in this study were female (63.5%). This trend was observed in other research with similar intervention mechanisms (31, 74, 75). Gender plays a role as a significant determinant of mental health (76). For example, women experience certain mental illnesses such as depression and anxiety at higher rates than men (77). Additionally, women are more likely to experience or suffer from violence as well as rape or attempted rape in their lifetime (76). The higher participation rate of women in this study might reflect this burden and need for services, however further research is needed. Additionally, further research on gender and the uptake and effectiveness of mobile technologies is needed.

### Survey Completion

Survey participation rates observed a drop from baseline at 48.3% to only 10.7% by the 6-months survey. The response rate for the baseline survey reflects the rates recorded in other studies. For example, a meta-analysis from (78), which examined response rates for online psychological surveys focused on adults with depression and/or general anxiety disorder, observed a mean response rate of ~43% (78). Another meta-analysis from 2008 demonstrated lower response rates, of 34% for online surveys, vs. 45% for paper surveys (79).

Regardless of the greater adoption of the internet among the general population, response rates for online surveys are observed generally to have remained low (80). In fact, survey response rates to online surveys are estimated to be 10% lower than surveys offered through other mediums, including paper surveys distributed by mail, and/or telephone surveys (81). Factors contributing to variability in response rates are believed to include population groups (employees in a company vs. the general population), socio-demographic factors (access to and literacy of technology, age, race), and personality types (conscientiousness, agreeability, and openness) (81). Another factor attributed to low response rates is the potential influence of “over surveying” populations (82).

Based on the analysis of reviews summarizing effective strategies for improved survey administration, health services may wish to incorporate the several strategies to survey delivery and design to improve response and engagement rates, which would improve future evaluative efforts and in turn future service delivery. These strategies include piloting surveys with a sample group prior to wider dissemination, administering surveys using different online mediums and techniques (e.g., text messaging, emails, phone prompts, etc.), ensuring ease of accessibility and appropriateness for population of interest in survey design to accommodate respondent comfort level and literacy, and limit survey size to increase likelihood of survey completion (78, 81).

### Barriers to Care

Identifying barriers to care is pivotal in the development and design of effective and efficient services to support the health and well-being of individuals with addiction and mental health challenges. The top, most frequently reported perceived barriers to care identified in this study were costs of services, not knowing how to access services, not knowing what services were available, and stigma with accessing services.

“Costs of services” is reflected in several larger powered studies as being a top barrier for individuals in Canada as well as the United States seeking to accessing services and treatment (6, 83). Although Canada is known for its universal health care system, health care within Canada does not fully cover certain forms of needed substance use and mental health services such as psychotherapy (84), and does not provide coverage for all pharmaceutical prescriptions (85, 86). Instead, its focus is to address acute mental health service needs of severe presentations of addiction and mental health (87). Therefore, individuals who have mild to moderate addiction and/or mental health issues frequently must pay for services out of pocket (87). However, it is estimated that the medical coverage for 1 in 5 Canadians do not cover pharmaceuticals, and 1 in 10 cannot afford to pay out of pocket (85).

Not knowing how to access services was the second most frequently reported barrier amongst respondents in this study. Reflecting on higher power studies such as the 2014 GapMap, which included a population survey of ~6,000 adults from Alberta, “didn't know where to get help” was reported by over a quarter of respondents (27.7%), and was the fourth most frequently reported barrier (6). Another Canadian study analyzing unmet need for addiction and/or mental health issues also identified this barrier as the fourth most frequently reported barrier at 15.5% of respondents (88). Variability observed in reflection of this and other barriers to care are likely the result of the differing survey methodologies, geographic regions, and population groups.

Literature providing further analysis on the lack of knowledge around what services are available as a barrier to addiction and mental health care is limited (88), and therefore deeper reflection on this factor using higher power studies was not possible. However, where noted in the literature, “not knowing what services were available” was grouped in as a barrier of “personal circumstance” vs. “feature of the health care system,” along with “haven't gotten around to it yet” or “job interfered,” potentially suggesting that the onus is on the individual vs. the health system to find solutions to said barrier (89, 90). This is perhaps tied to issues around cost coverage, as not all services and treatments are covered by Canada's Universal health care system, and/or reflects systemic stigma, where the expectation is for individuals suffering from mental health issues to be independently motivated enough to self-identify solutions, instead of health systems proactively reaching out to share service information in a proactive manner and/or accepting responsibility for the navigation of patients when services may cross-pollinate across public and private sector lines.

Although stigma with accessing services was noted frequently as a top barrier across all respondent groups, it was higher than average for individuals with less stable housing, as well as Indigenous and African/Caribbean individuals. Systemic prejudice and racism are reflected in the literature as being a significant barrier to care (87, 91). For example, there are identified system biases to process someone through intake, including in the physical, linguistic, and geographic accessibility of centers and services (e.g., assumptions that transit to a facility would not be a problem, intake forms are written at an appropriate reading level for a wide audience including accommodating newcomers, that individuals are able to record and monitor time with ease, etc.) (87). “Factors including race, class, socioeconomic status, ability/disability, gender identity, sexual orientation, citizenship status, and mental health diagnosis influence people's experience accessing services which affect their ability to receive support. Experiencing multiple barriers creates an intersectionality that heighten marginalization and creates difficulty accessing services (91).”

Attitudinal barriers to care (e.g., the desire to self-manage addiction and/or mental health issues), were not questioned in this survey. This is likely because participants in the study were actively seeking access to services, and thus it could be implied that they were not choosing to self-manage their issue at the time of enrolment. Nevertheless, it is important to highlight that the attitudinal barrier of preference to self-manage has been identified as a primary barrier to care in other population-based surveys (6, 92).

### COVID-19

Canadians have been socially isolating and distancing themselves from others to assist in the reduction of transmission of COVID-19. This has resulted in many individuals working from home, as well as a demand for access to virtual services. During this unprecedented time, digital health innovations provide opportunities to bridge existing and anticipated gaps in mental health care, providing solutions to connect services with individuals in their homes or where they are, on demand.

Additionally, the pandemic has provided an opportunity for the expedited uptake of new technologies. Although historically slow on the uptake of virtual service options, health systems have been forced to quickly initiated the use of virtual care in order to reach patients in isolation (93). This adaptation of the system to appease public health restrictions presents significant opportunity for ongoing service provision via online or virtual mediums post-pandemic, as the use of digital health interventions become more commonly used and palatable to what is otherwise a cautious public system.

Alternatively, cautions must also be raised. Gaps in the provision of care creates the incentive for individuals to seek support from private enterprises. There is general agreement among ethicists, legal professionals and researchers that domestic and global legal and regulatory bodies have not been able to keep up with the proliferation of novel innovations, and as a result existing legal and regulatory infrastructure are grossly inadequate (94–97). This lack of adequate legal and regulatory protection exposes the public to risks from digital products including unvalidated claims, which is particularly concerning as it may divert individuals from accessing effective treatment for serious health conditions.

Therefore, in order to keep up with demand and address gaps in service in a post-pandemic context, public health systems need to expedite the uptake of novel interventions and services in a rigorous manner, while legal and regulatory bodies update and correct frameworks to protect the general public from unfounded or unsafe technologies for private purchase.

### Additional Considerations

Although digital interventions have the potential to enhance and expand service provision significantly, caution is warranted to ensure that any foreseeable unintended repercussions are carefully mapped, as well as strategically and proactively addressed in order to mitigate any potential harm to individuals.

An important complexity which must be considered in the development of technologies for mental health is the intersection between technology to support mental health and behavioral addictions to technology (98). For example, a 2013 systematic review identified a strong correlation between Attention Deficit Hyperactivity Disorder (ADHD) and Internet Gaming Disorder (99). Technological interventions, including the receipt of supportive daily text messages, may result in positive reinforcement of an individual's general screen time, which could potentially aggravate existing issues for some individuals actively managing and/or who are vulnerable to certain mental illnesses associated with technology. It is important then for technological interventions for mental health to be delivered in reflection of any preexisting mental health related illnesses, to ensure interventions support rather than harm an individual's mental well-being.

### Limitations

As noted, this study had several limitations, including low sample size. Given that the sample size of our study was considerably less that the estimated sample size of 686 needed to detect differences, our study did not achieve the desired power of 80%. This notwithstanding the low sample size, our study was able to detect statistically significant differences in the CORE-10 mean scores from baseline to 12 weeks and 6 months.

Additionally, this study was subject to selection bias, as participation in the program and completion of surveys was voluntary and complimentary to existing care. Therefore, individuals who enrolled into the Text4Support program may not be reflective of the total population of Alberta, or of individuals seeking care at these clinics, as they may have greater eagerness to engage in certain forms of treatment than others accessing the site. For example, female gender (63.5%) seems to be overrepresented in our sample and is not elective of the wither the proportion of the Albert's population or of these seeking services in the clinic. It is possible that females are more likely to subscribe to text-based support services or to complete online surveys. In the user satisfaction surveys for the Text4Mood and Text4Hope programs, over 80% of survey respondents were female (31, 74, 75).

Another limitation of this study is that clinical outcome scores were subject to various confounding variables as highlighted, including the potential mental health impact of the COVID-19 pandemic, as well as the potential that individuals in the program received access to other mental health treatments/services in conjunction with this intervention. Additionally, participants may have had comorbid addiction and mental health issues, for which text messaging on other themes may have influenced participant responses to survey questions and/or clinical outcome scores.

The Text4Support baseline survey included several questions assessing an individuals' level of personal support. These questions were not repeated in 12-weeks and 6-months surveys. As text messaging has been found to serve as a connecting mechanism to an individual's support network (17, 100), it would have been interesting to examine whether an individual's perceived level of support changed over the course of the intervention, which may have impacted their reported CORE scores in follow up surveys.

## Conclusions

We are in the midst of a digital revolution, where what began with the emergence of the internet has evolved from comparatively simple digitization to the complex, globally-integrated networks and systems we observe today. These systems are restructuring and disrupting governance and economics frameworks, and are so exponentially transformative they are forcing us to reconsider our basic conceptualization of what it means to be alive (101).

With the global pandemic restricting the movement of individuals, the acceptance of virtual care solutions for use by Canadian physicians has increased (102). Whether or not this change becomes sustained following COVID-19, remains to be seen. Nevertheless, technology proliferates quickly, with most Canadians now armed with various forms of mobile technology and connected instantaneously to online networks and platforms. Canada has long been at a point where existing service provision is grossly underwhelming the expectations and needs of Canadians, and we now have an opportunity to propel our systems into the 21st century through the greater adoption of evidence-supported digital technology to complement and enrich care.

This study provided a preliminary analysis of the efficacy of a supportive text messaging intervention seeking to support the mental health and well-being of individuals accessing mental health and addiction services at two mental health and addiction clinics. The findings from this study, albeit anecdotal due to low sample size, provide interesting insights that appear to reflect the findings of other higher-powered studies. Taken together, this study suggests that, at minimum, supportive text messaging offers a viable tool to complement existing programs, as well as to increase patient-system contact for patients seeking support for addiction and mental health concerns.

## Data Availability Statement

The raw data supporting the conclusions of this article will be made available by the authors, without undue reservation.

## Ethics Statement

The studies involving human participants were reviewed and approved by Health Research Ethics Board—Health Panel (PRO00086163). The patients/participants provided their written informed consent to participate in this study.

## Author Contributions

JN, WV, and AG contributed to study design, manuscript drafting, revision of manuscript for critical content, data analysis and interpretation, and approved the final version for publication. SS and LU revision of manuscript for critical content, data analysis and interpretation, and approved the final version for publication. VA contributed to study conception and design, manuscript drafting, revision of manuscript for critical content, data analysis and interpretation, and approved the final version for publication. All authors contributed to the article and approved the submitted version.

## Conflict of Interest

The authors declare that the research was conducted in the absence of any commercial or financial relationships that could be construed as a potential conflict of interest.
